# Supplementary material for: Structural evolution during inverse vulcanization
Source: Nat Commun. 2024 Jun 29;15:5507. doi: 10.1038/s41467-024-49374-y (PMC11217493; doi:10.1038/s41467-024-49374-y)
Supplement: Supplementary file 1 — Supplementary Information [file 41467_2024_49374_MOESM1_ESM.pdf]

Supplementary Information

Structural evolution during inverse vulcanization

Botuo Zheng[#, a], Liling Zhong[#, a], Xiaoxiao Wang[a], Peiyao Lin[a], Zezhou Yang[a], Tianwen Bai*[, b], Hang, Shen*[, c], Huagui Zhang *[, a]

[a] College of Chemistry and Materials Science, Fujian Key Laboratory of Polymer Materials, Fujian Normal University, Fuzhou 350007, China

[b] Key Laboratory of Medical Electronics and Digital Health of Zhejiang Province in Jiaxing University, College of Biological, Chemical Sciences and Engineering, Jiaxing University, Jiaxing 314001, China

[c] College of Materials and Chemical Engineering, Minjiang University, Fuzhou 350108, China

Catalogues

Supplementary Fig. 1. A typical inverse vulcanization curves..4

Supplementary Table 1. Monomer stuructures and polymerization conditions of the inverse vulcanization samples investigated by rheological tests 4

Supplementary Fig. 2. Strain sweep result of DIBS1-1 5

Supplementary Fig. 3. Strain sweep result of DIBS1-2 5

Supplementary Fig. 4. Strain sweep result of DIBS1-3 5

Supplementary Fig. 5. Strain sweep result of DIBS1-4 6

Supplementary Fig. 6. Frequency sweep results of DIBS1-1 6

Supplementary Fig. 7. Frequency sweep results of DIBS1-2 7

Supplementary Fig. 8. Frequency sweep results of DIBS1-3 7

Supplementary Fig. 9. Frequency sweep results of DIBS1-4 8

Supplementary Table 2. G' retrieved from frequency sweep curves compared with G' obtained from the sampling times. 8

Supplementary Fig. 10. Correlation between b_T and ΔT for DIBS1 sample..9

Supplementary Table 3. Coordination number (z_{co}) of DIBS calculated from T_g and M_c. 10

Supplementary Fig. 11. XPS spectra of S 2*p* signals of DIBS1 sample..12

Supplementary Fig. 12. Sulfur proportion and C/H ratio of inverse vulcanization products..........13

Supplementary Fig. 13. Time sweep result of DIBS2 sample..14

Supplementary Fig. 14. Time sweep result of DIBS3 sample..14

Supplementary Fig. 15. Time sweep result of DIBS4 sample..15

Supplementary Fig. 16. The time sweep result of DIBS5 sample..15

Supplementary Fig. 17. Time sweep result and the photo of DVBS2 sample 15

Supplementary Fig. 18. Time sweep result of DVBS1 sample..16

Supplementary Fig. 19. Time sweep result of SOS1 sample 16

Supplementary Fig. 20. Time sweep result of SOS2 sample 16

Supplementary Fig. 21. Time sweep result of SOS3 sample 17

Supplementary Fig. 22. Time sweep result of SOS5 sample 17

Supplementary Fig. 23. Frequency sweep results of SOS4-1 sample..18

Supplementary Fig. 24. Frequency sweep results of SOS4-2 sample 18

Supplementary Fig. 25. Frequency sweep results of SOS4-3 sample 19

Supplementary Fig. 26. Frequency sweep results of SOS4-4 sample 19

Supplementary Fig. 27. Correlation between b_T and ΔT for SOS4 sample 20

Supplementary Fig. 28. XPS spectra of S 2*p* signals of SOS4 sample 20

Supplementary Fig. 29. Time sweep result of DCPD1 sample 21

Supplementary Fig. 30. Time sweep result of DCPD2 sample 21

Supplementary Fig. 31. Time sweep result of DCPD3 sample 22

Supplementary Fig. 32. Time sweep result of DCPD4 sample 22

Supplementary Fig. 33. The high-resolution S 2*p* XPS spectra of poly(DCPD-*r*-S) 23

Supplementary Table 4. Inverse vulcanization of mono-alkenes and an aliphatic ester for mechanistic investigation. 24

Supplementary Fig. 34. ^{1}H NMR spectrum of byproduct 1,2-dithiol-2-phenyl-3-thione 25

Supplementary Fig. 35. ^{13}C NMR spectrum of byproduct 4-phenyl-1,2-dithiol-3-thione 25

Supplementary Fig. 36. HMBC spectrum of byproduct 4-phenyl-1,2-dithiol-3-thione 26

Supplementary Fig. 37. HSQC spectrum of αMSt samples 27

Supplementary Fig. 38. ^{1}H NMR and HMBC spectra of the soluble fraction of DIBS1-4 27

Supplementary Fig. 39. ^{1}H NMR spectra of 4-MStS sample 28

Supplementary Fig. 40. HMBC spectra of 4-MStS sample 28

Supplementary Fig. 41. ^{1}H NMR spectrum of APES sample 29

Supplementary Fig. 42. ^{1}H NMR spectrum of EODS sample 29

Supplementary Fig. 43. HMBC spectrum of EODS sample 30

Supplementary Fig. 44. ^{1}H NMR spectrum of P(S-SA) 30

Supplementary Fig. 45. ^{1}H NMR spectrum of soluble fraction of SOS4-4 sample 31

Supplementary Fig. 46. HMBC spectrum of soluble fraction of SOS4-4 sample 31

Supplementary Fig. 47. FT-IR spectrum of soluble fraction of SOS4 sample 32

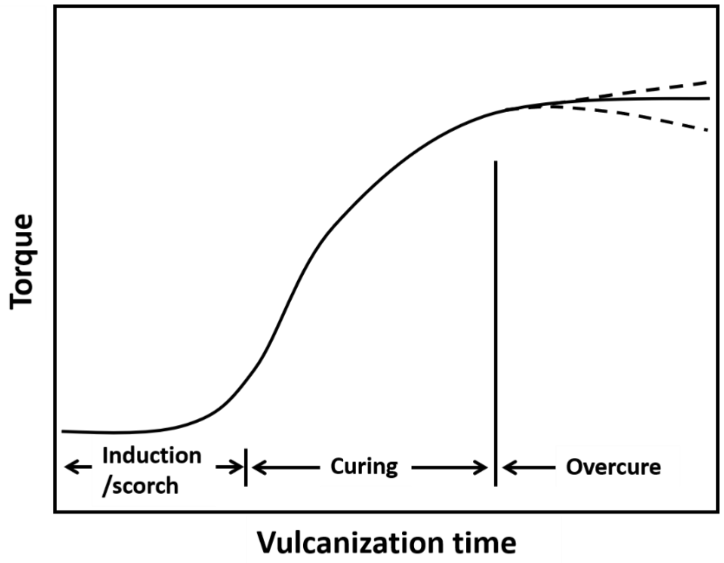

Supplementary Fig. 1 A typical inverse vulcanization curve including three stages in different patterns. [1]

Supplementary Table 1. The inverse vulcanization of DIB, DVB, SOS and DCPD for rheological investigation.

Sample	Monomer structure	S_8 (g)	Monomer (g)	Sulfur feed ratio[a]	Temperature (°C)	Gelation time (min)	Test time (min)
DIBS1	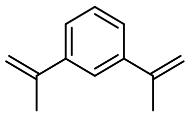 1,3-diisopropenylbenzene(DIB)	0.91	1.10	2	160	54	265
DIBS2		0.91	1.13	2	170	28	202
DIBS3		0.90	1.11	2	180	13	49
DIBS4		1.11	0.91	3	160	14	255
DIBS5		1.24	0.76	4	160	11	165
DVBS1	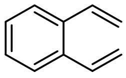 Divinylbenzene(DVB)	0.99	1.01	2	160	11	243
DVBS2					180	7	256
SOS1	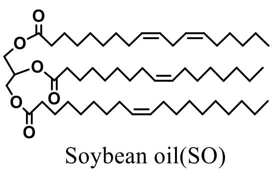 Soybean oil(SO)	0.73	1.27	4	160	77	268
SOS2		0.94	1.08	6	160	58	262
SOS3		0.73	1.27	4	170	30	254
SOS4		0.73	1.27	4	180	14	250
SOS5		0.94	1.08	6	180	12	249
DCPDS1	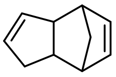 Dicyclopentadiene(DCPD)	0.98	1.02	2	160	60	267
DCPDS2		0.99	1.01	2	170	18	256
DCPDS3		0.99	1.01	2	180	7	139
DCPDS4		1.32	0.68	4	160	50	231

[a] The sulfur feed ratio is the molar ratio of sulfur over alkene groups ([S]/[C=C]) in the feedstocks.

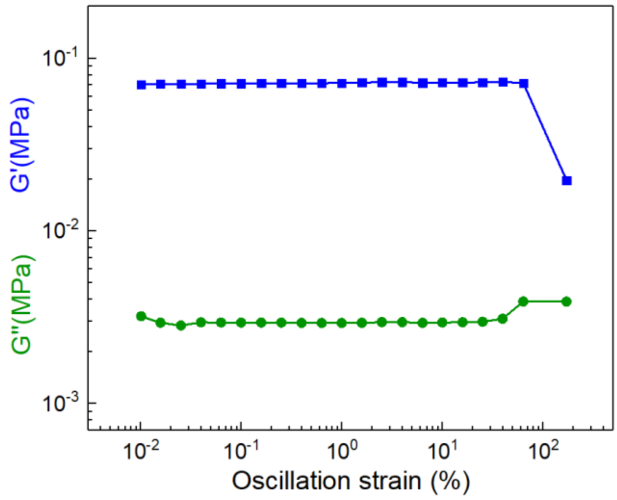

Supplementary Fig. 2 Strain sweep result of DIBS1-1 sample at 10 rad/s and 110 °C.

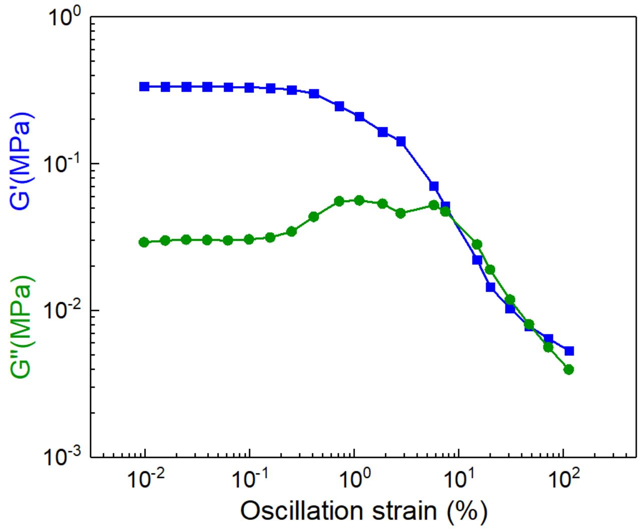

Supplementary Fig. 3 Strain sweep result of DIBS1-2 sample at 10 rad/s and 110 °C.

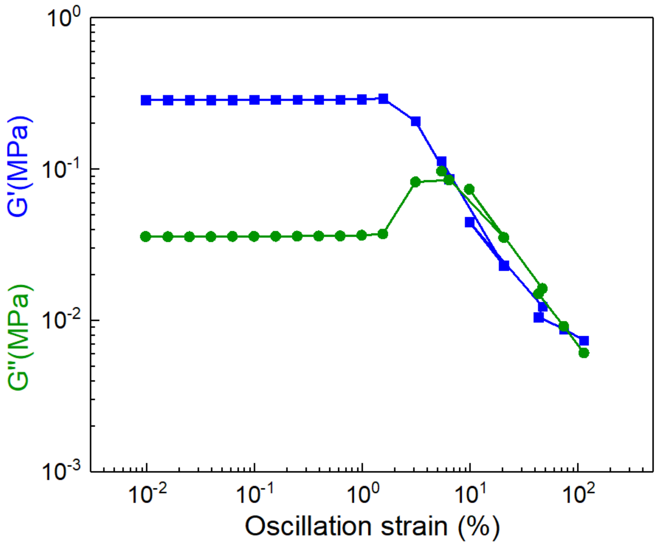

Supplementary Fig. 4 Strain sweep result of DIBS1-3 sample at 10 rad/s and 110 °C.

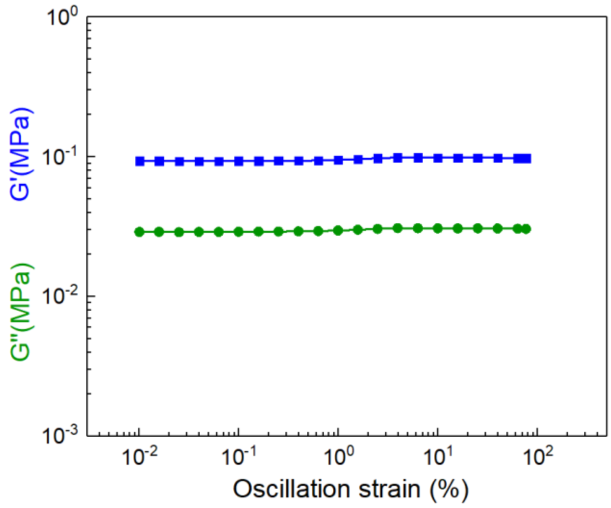

Supplementary Fig. 5 Strain sweep result of DIBS1-4 sample at 10 rad/s and 110 °C.

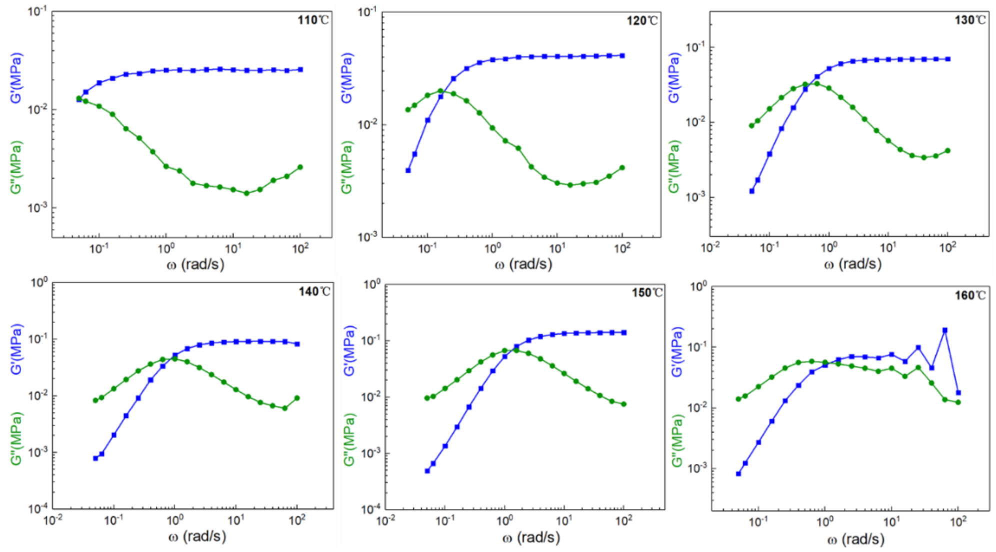

Supplementary Fig. 6 Frequency sweep results of DIBS1-1 sample at temperatures from 110 to 160 °C.

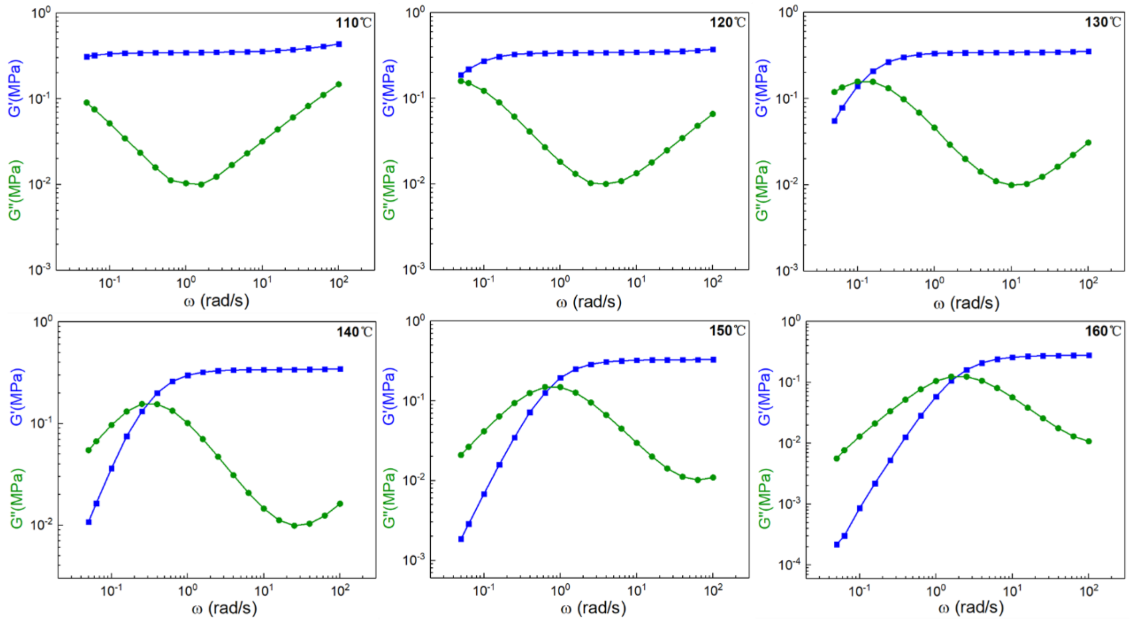

Supplementary Fig. 7 Frequency sweep results of DIBS1-2 sample at temperatures from 110 to 160 °C.

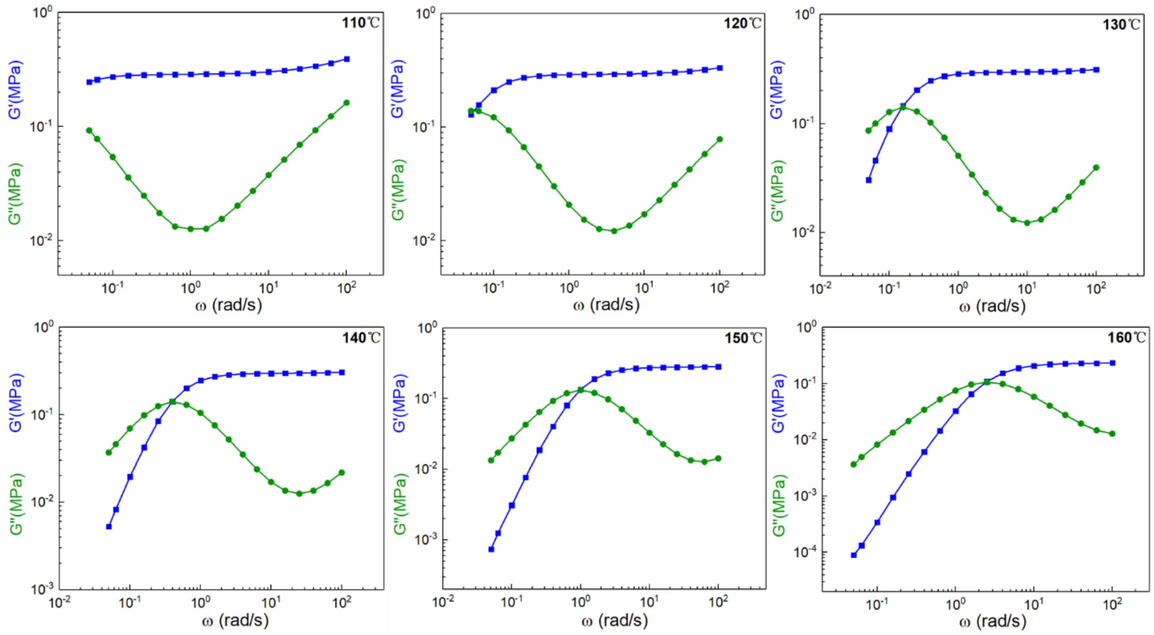

Supplementary Fig. 8 Frequency sweep results of DIBS1-3 sample at temperatures from 110 to 160 °C.

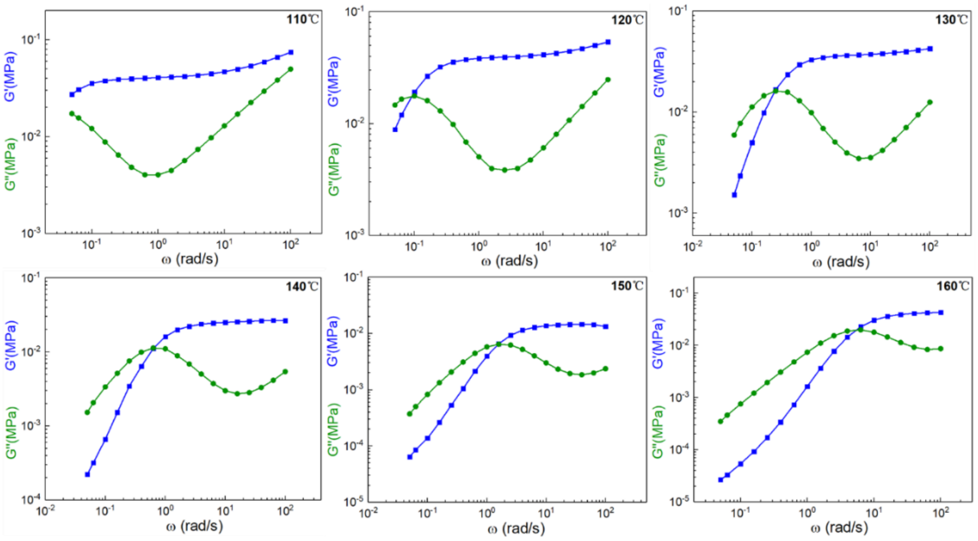

Supplementary Fig. 9 Frequency sweep results of DIBS1-4 sample at temperatures from 110 to 160 °C.

Supplementary Table 2. The G' at 1 rad/s is retrieved from frequency sweep curves at 160 °C to compare with G' obtained from G'-t curve at the sampling times.

Sample	G'^{a} (MPa)	G'^{b} (MPa)
DIBS1-1	1.96×10^{-2}	5.09×10^{-2}
DIBS1-2	5.63×10^{-2}	5.86×10^{-2}
DIBS1-3	1.21×10^{-3}	3.25×10^{-2}
DIBS1-4	6.84×10^{-6}	2.61×10^{-3}
SOS4-1	2.45×10^{-3}	1.66×10^{-3}
SOS4-2	1.64×10^{-3}	4.53×10^{-2}
SOS4-3	5.55×10^{-4}	3.13×10^{-2}
SOS4-4	8.96×10^{-4}	2.80×10^{-2}

[a] The G' obtained from G'-t curve at the sampling times.

[b] The G' at 0.2% and 1 rad/s is retrieved from frequency sweep curves at 160 °C.

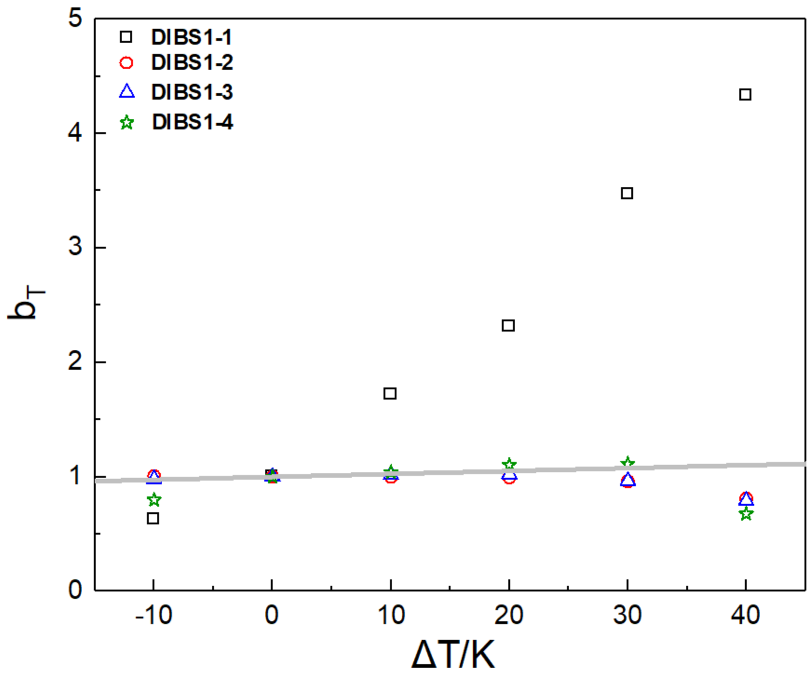

Supplementary Fig. 10 Correlation between b_T and ΔT for DIBS1-1, DIBS1-2, DIBS1-3, and DIBS1-4. Symbols represent the experimental data. Gray lines are given by T/T_r.

The vertical shifting seems that the most plausible concept is based on the Rouse model, where the complex modulus is scaled with the temperature and mass density as follows[2]:

$$b_T = \frac{T\rho(T)}{T_r\rho(T_r)} \tag{1}$$

where $\rho(T)$ and $\rho(T_r)$ are the mass densities at T and T_r (the reference temperature in TTS), respectively. Supplementary Fig. 10 shows the correlation between b_T and $T - T_r$ (ΔT) for DIBS1-1, DIBS1-2, DIBS1-3, and DIBS1-4. The grey line depicts the theoretical b_T depending on T when density of the sample $\rho(T)$ is a constant taking equation (1). The overlap of experimental b_T and grey line indicates the density of DIBS samples (DIBS1-2, DIBS1-3 and DIB1-4) can be regarded as a constant independent on the temperatures as long as the temperature is lower than 160 °C.

Calculating coordination number z_{co} of polymer segment from T_g and M_c

According to the reference[3], the z_{co} represents the covalent bond connected to the unit beads, which is to be calculated. The critical volume fraction ϕ_c, below which the nonaffinity deformation will significantly result in the loss of modulus, can be expressed by z_{co} as $\phi_c = \phi_c^* - \Lambda z_{co}$where ϕ_c^* is the packing fraction in the limit z_{co} = 0. On the other hand, ϕ_c can be expressed as $\phi_c = \exp{(-\alpha_T T_c - C)}$, where α_T is thermal expansion coefficient and T_c is the critical temperature, i.e., glass transition temperature T_g in polymers. By expanding the exponential to linear order using Taylor formula and omit high order terms, one has $\phi_c = 1 - \alpha_T T_g - C$. With two equations involving φ_c, we obtain the relationship between T_c and z_{co}as follows:

$$\alpha_T T_g = 1 - C - \phi_c^* + z_{co}\Lambda \quad (2)$$

Where $\Lambda \approx 0.1, C \approx 0.48$ pointed out by the literature[3] considering similarity of DIB to styrene. For a random packing of hard spherical polymer beads, $\phi_c^* \approx 0.64$. Most polymers have a α_T ranging from 2×10^{-4} to 4×10^{-4} K^{-1} and an average of 3×10^{-4} K^{-1} is adopted in this study[4]. By substituting the values into the equation, we have:

$$z_{co} = \frac{T_g}{3\times10^{-3}} + 1.2 \quad (3)$$

Thereby, the z_{co} of samples DIB-S can be calculated from T_g, which is listed in column $z_{co,T}$ of Supplementary Table 3:

Supplementary Table 3. $z_{co}$of DIBS samples calculated from T_g and M_c.

Sample	T_g (K)	$z_{co,T}$ ^{a}	M_c (kDa)	$z_{co,M}$ ^{b}
DIBS1-1	283	2.049	93.2	2.011
DIBS1-2	308	2.124	13.4	2.075
DIBS1-3	312	2.136	14.8	2.067
DIBS1-4	309	2.127	47.9	2.021

[a] As determined from T_g. [b] As determined from M_c.

The coordination number can also be estimated from molecular weight between crosslinks (M_c, Table 3). The DIBSs are crosslinked homogenous networks, therefore has infinite molecular weights. By assuming the molecular weight of Kuhn segment (M_k) as 1 kDa [5], the $z_{co,M}$ can be calculated from the number of Kuhn segments as beads between crosslinks ($n_k = M_c/M_k$) taking the

coordination number of linear beads and crosslinked beads as 2 and 3, respectively:

$$z_{co,M} = \frac{2(n_k-1)+3}{n_k} \quad (4)$$

The calculation result is showed in Supplementary Table 3. Clearly the z_{co} calculated from T_g and M_c has similar trend of increase in curing followed by decrease in over-cure stage, although the discrepancy in values due to relatively arbitrary α_T and M_k used which surely differed as the unit structures evolved. Anyway, the calculation revealed that the decline in T_g in the over-cure stage is likely to arise from the loss of network connectivity.

As for poly(SO-*r*-S) (SOS), the parameters (Λ, C, M_k *and* α_T) of SOS samples which have entanglement and branch structures as well as unreacted alkenyl group simultaneously, should be distinct from those of polystyrene studied by the reference. Therefore, the z_{co} of SOS is hard to be estimated by the same algorithm, which requires further investigation.

XPS of DIBS

The sulfur rank can be estimated from the integral ratios, defined as SCR, between S 2*p* signal of sulfur atom among sulfur chain (S-S) and sulfur atom connected to carbon. The scr. Since in most cases there is two sulfur atoms at two end of a sulfur chains between organic units, the sulfur rank can be estimated to be SCR×2+2. The latter term denotes two sulfur atoms connected to carbon, and the former term denotes the number of atom in the mid of the sulfur chain.

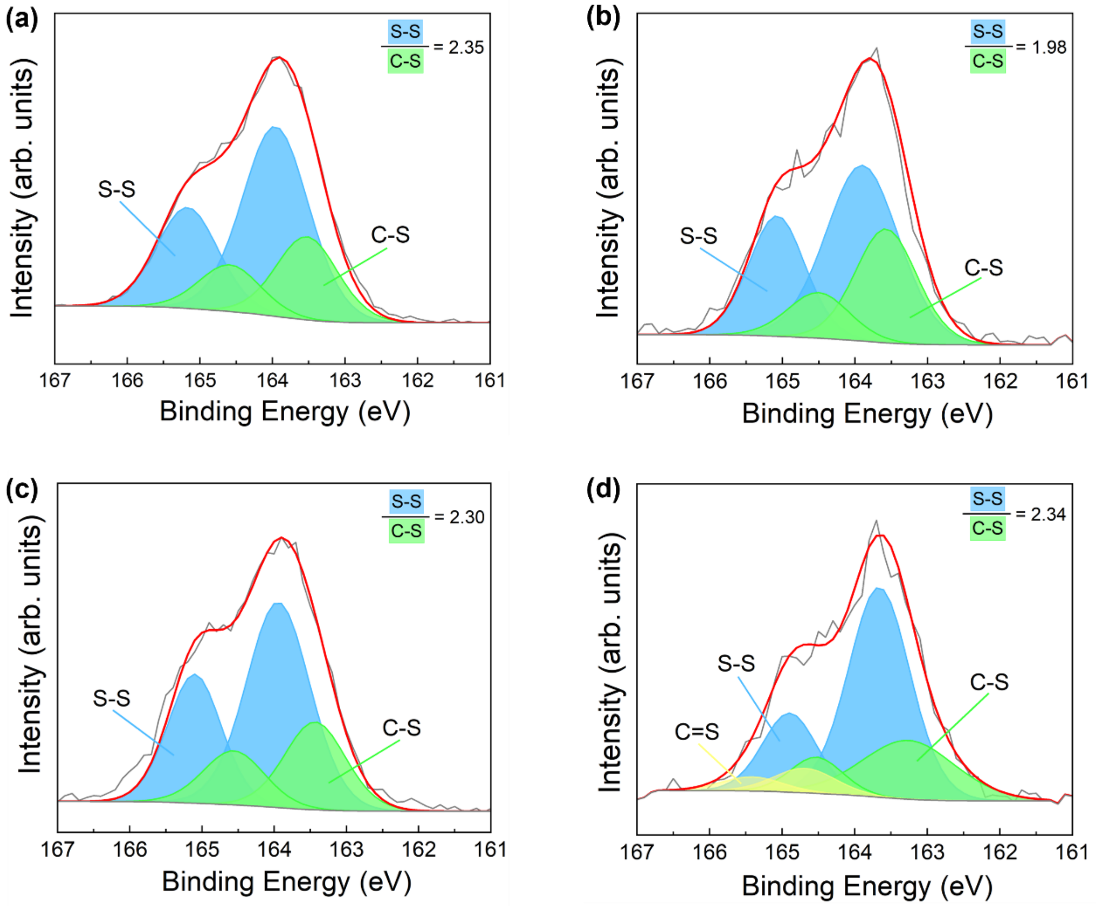

Supplementary Fig. 11 XPS spectra of S 2*p* signals of DIBS1. (a) DIBS1-1; (b) DIBS1-2; (c) DIBS1-3; (d) DIBS1-4.

Element Analysis (CHNS)

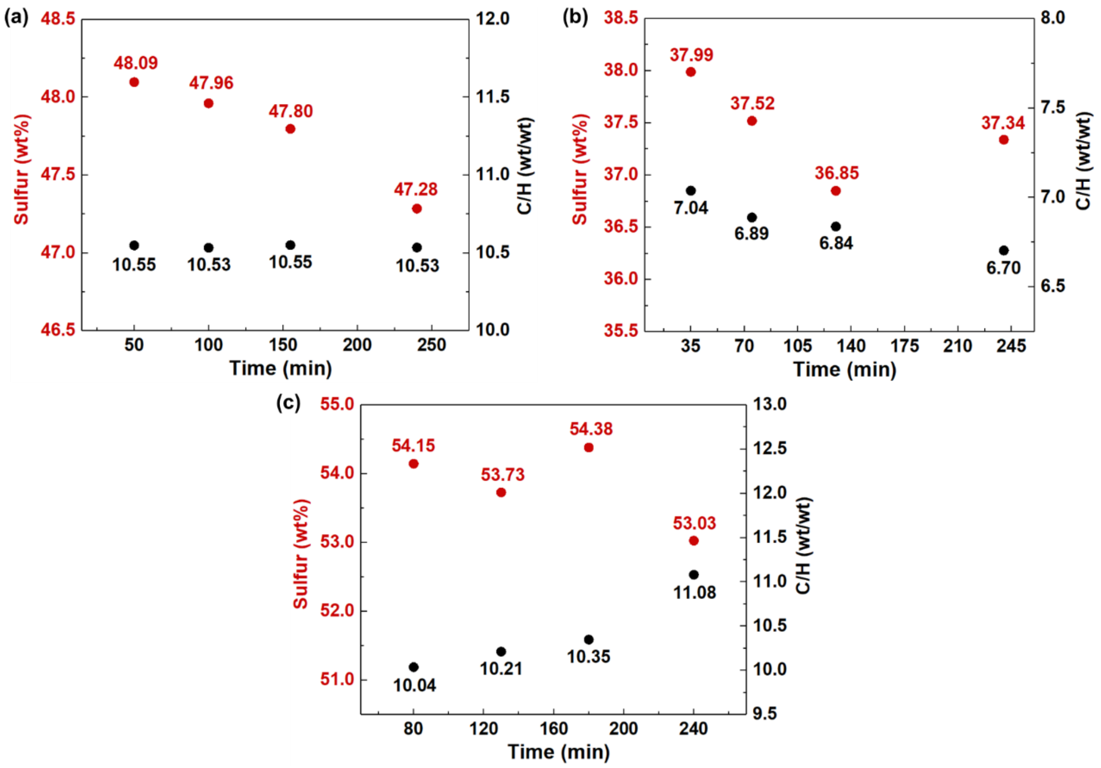

Supplementary Fig. 12 Sulfur proportion and C/H ratio of inverse vulcanization products of (a) DIB, (b) SO and (c) DCPD presented as at different reaction time determined by element analysis (CHNS).

Composition of the samples at different stages (DIBS1, SOS4 and poly(S-DCPD)) are determined by element analysis. The C/H and sulfur proportion is calculated and plotted against reaction time as Supplementary Fig. 12. Theoretically, the generation of H_2S during inverse vulcanization would lead to a decrease in sulfur proportion and an increase in C/H ratio. As the figure shows, the sulfur proportion continually decline (48.1% to 47.2%) in DIBS as inverse vulcanization proceeds in contrast to the fluctuation of C/H ratio (10.55 to 10.53). The result suggests the release of sulfur-rich species including organosulfides besides H_2S during inverse vulcanization. The organsulfides generated from side reaction with relatively low boiling points evaporated from the reaction mixture leading to loss of carbon simultaneously, which can be seen as yellow oily substance sticking on the wall of the reaction vessel. The similar trend is observed in elemental analysis results of SOS4 with a drop in sulfur proportion (38.0% to 36.9%) albeit the obvious decrease of C/H ratio (7.038 to 6.7). This can also be rationalized by evaporation of organosulfide side products beside H_2S loss, especially considering the SO is a bio-derived mixture containing impurity with low boiling points.

Lastly, the composition of inverse vulcanization products of DCPD experiences a drop in sulfur proportion (54.1% to 53.0%) accompanied with the increase in C/H ratio (10.0 to 11.1), demonstrating that the existence of H_2S loss but trace discharge of organosulfides in the stable DCPD-crosslinked polysulfides.

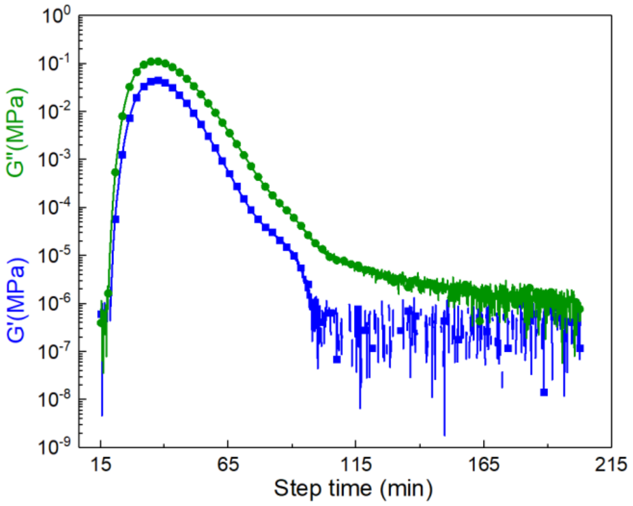

Supplementary Fig. 13 Time sweep result of DIBS2 sample with a sulfur feed ratio of 2 at 1 rad/s, 0.2% and 170 °C.

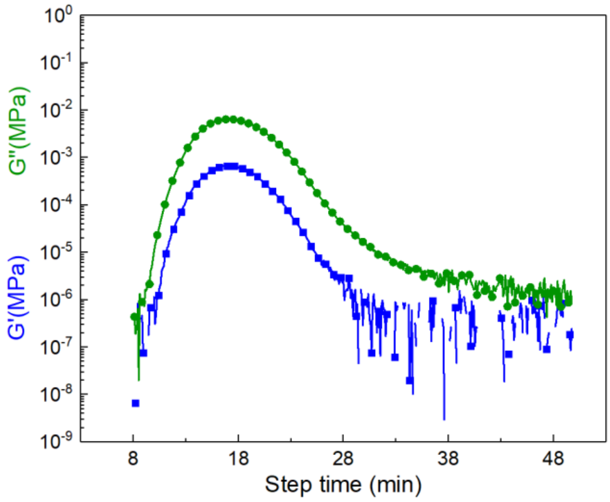

Supplementary Fig. 14 Time sweep result of DIBS3 sample with a sulfur feed ratio of 2 at 1 rad/s, 0.2% and 180 °C.

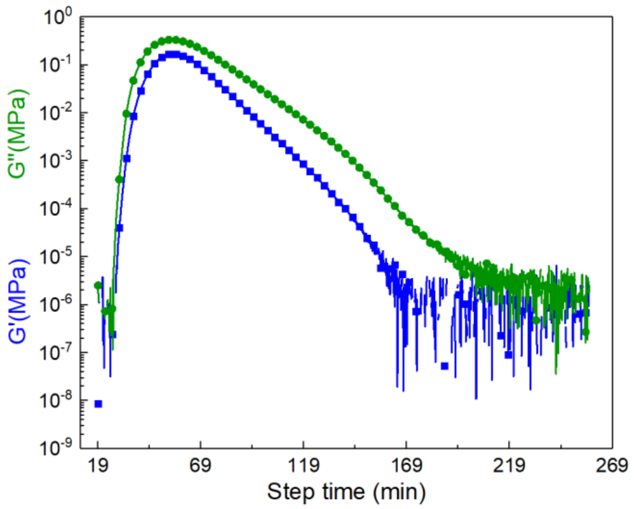

Supplementary Fig. 15 Time sweep result of DIBS4 sample with a sulfur feed ratio of 3 at 1 rad/s, 0.2% and 160 °C.

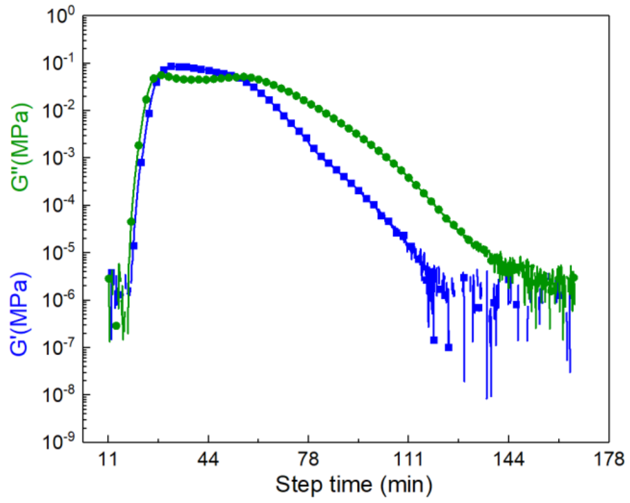

Supplementary Fig. 16 Time sweep result of DIBS5 sample with a sulfur feed ratio of 4 at 1 rad/s, 0.2% and 160 °C.

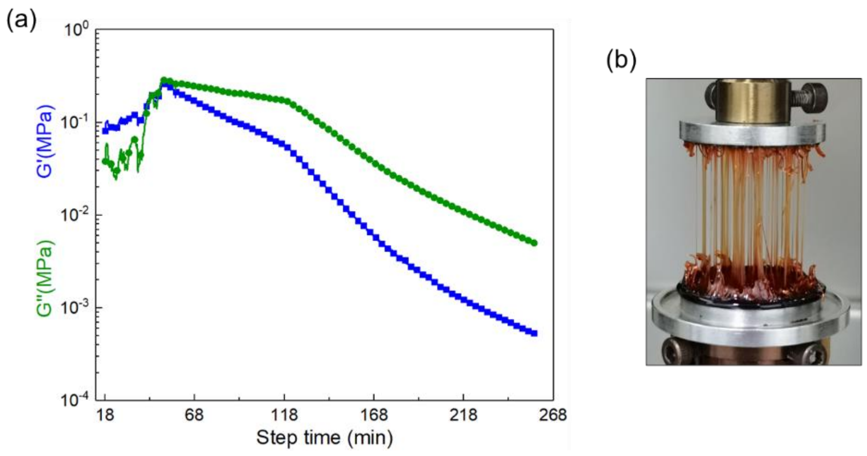

Supplementary Fig. 17 (a) Time sweep result of DVBS2 sample with a sulfur feed ratio of 2 at 1 rad/s, 0.2% and 180 °C. (b) The photo of DVBS2 sample in the rheometer after time sweep for 4 h.

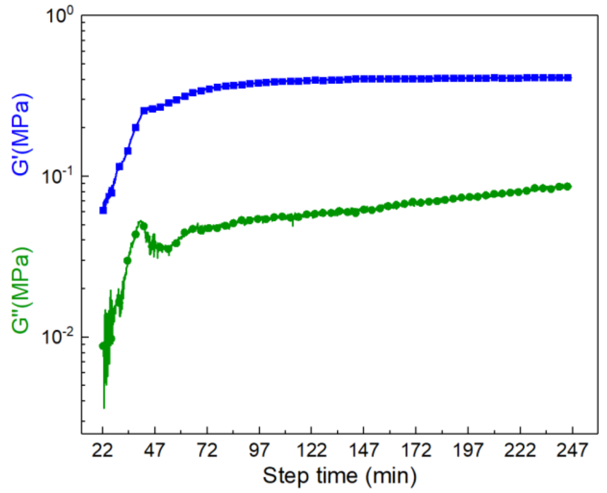

Supplementary Fig. 18 Time sweep result of DVBS1 sample with a sulfur feed ratio of 2 at 1 rad/s, 0.2% and 160 °C.

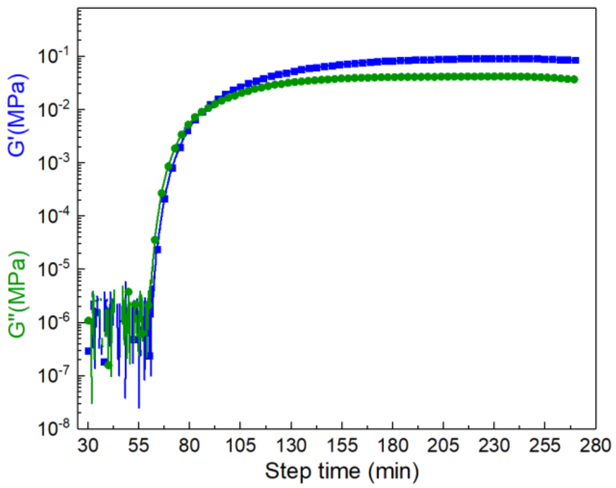

Supplementary Fig. 19 Time sweep result of SOS1 sample with a sulfur feed ratio of 4 at 1 rad/s, 0.2% and 160 °C.

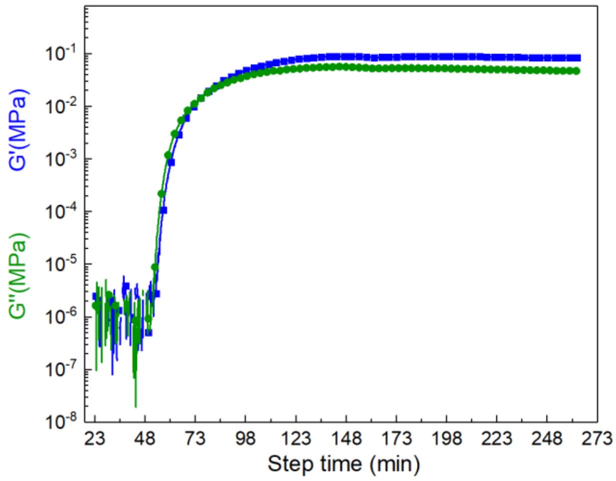

Supplementary Fig. 20 Time sweep result of SOS2 sample with a sulfur feed ratio of 6 at 1 rad/s, 0.2% and 160 °C.

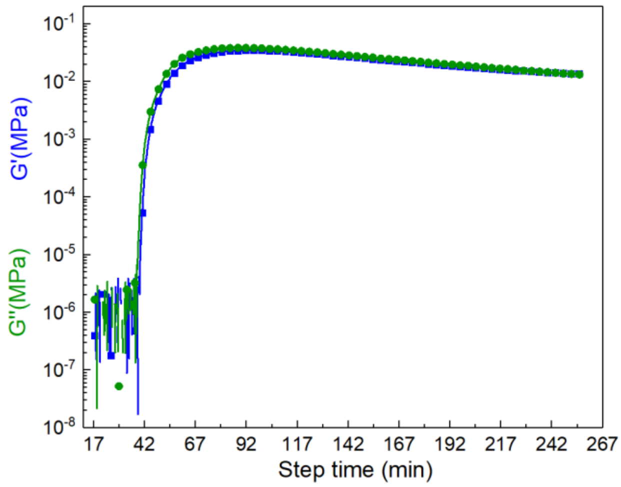

Supplementary Fig. 21 Time sweep result of SOS3 sample with a sulfur feed ratio of 4 at 1 rad/s, 0.2% and 170 °C.

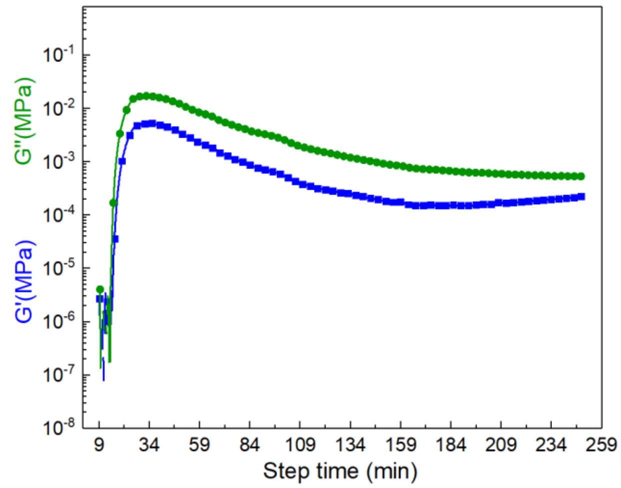

Supplementary Fig. 22 Time sweep results of SOS5 sample with a sulfur feed ratio of 6 at 1 rad/s, 0.2% and 180 °C.

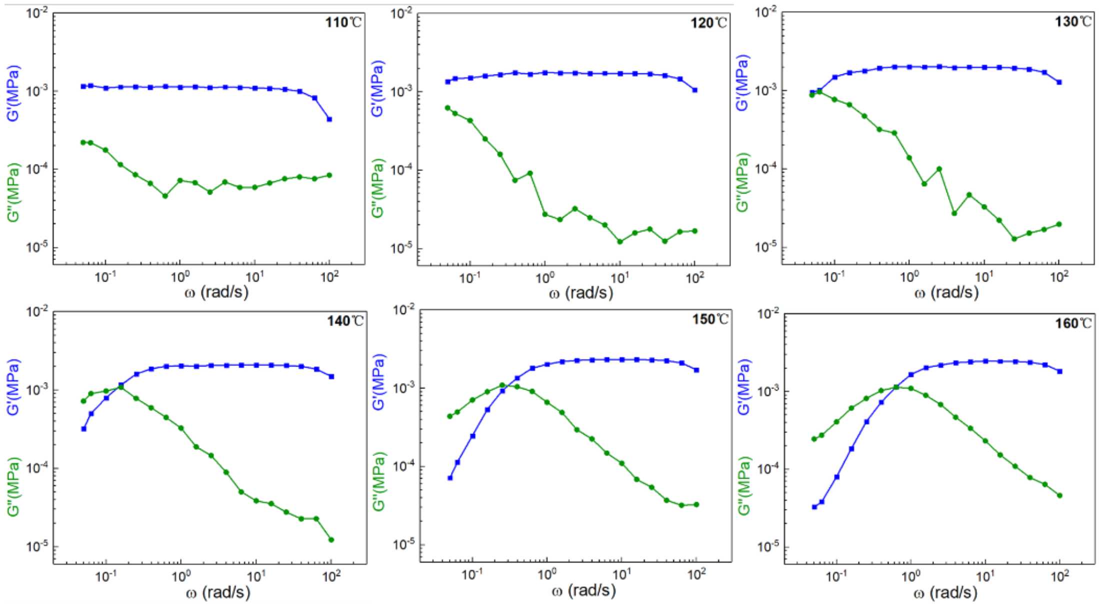

Supplementary Fig. 23 Frequency sweep results of SOS4-1 sample at temperatures from 110 to 160 °C.

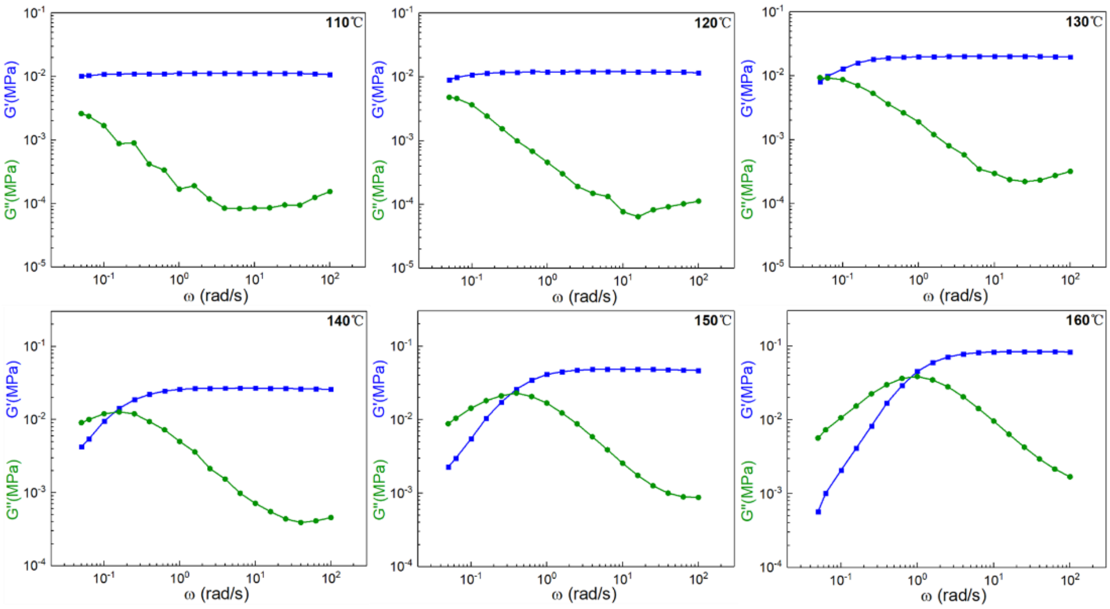

Supplementary Fig. 24 Frequency sweep results of SOS4-2 sample at temperatures from 110 to 160 °C.

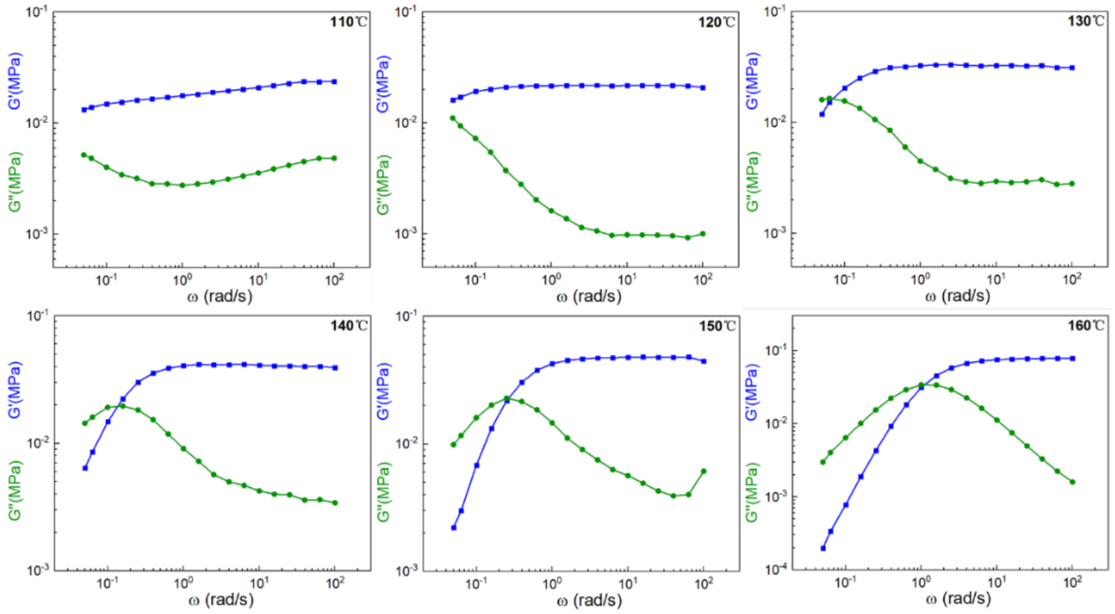

Supplementary Fig. 25 Frequency sweep results of SOS4-3 sample at temperatures from 110 to 160 °C.

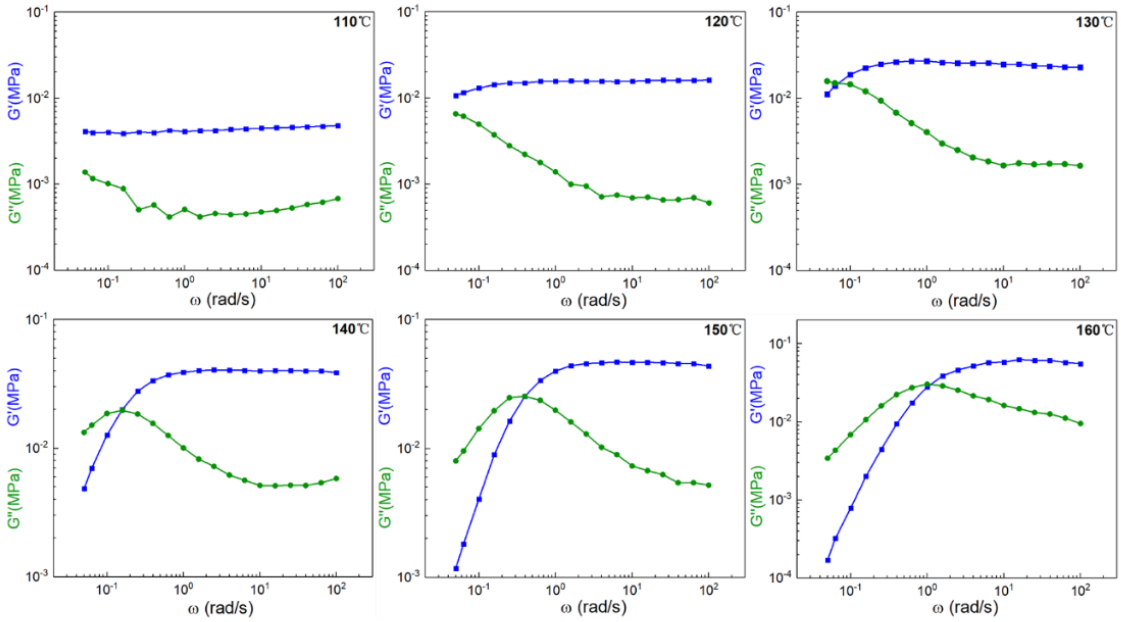

Supplementary Fig. 26 Frequency sweep results of SOS4-4 sample at temperatures from 110 to 160 °C.

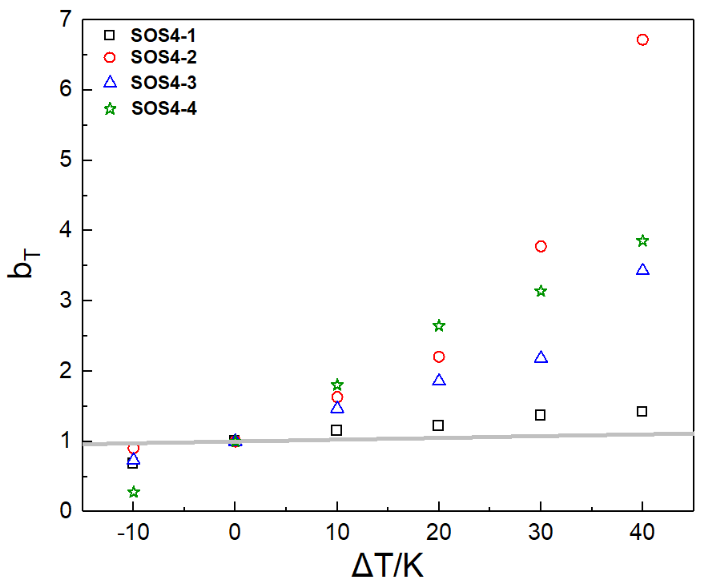

Supplementary Fig. 27 Correlation between b_T and ΔT for SOS4-1, SOS4-2, SOS4-3, and SOS4-4. Symbols represent the experimental data. Gray lines are given by T/T_r.

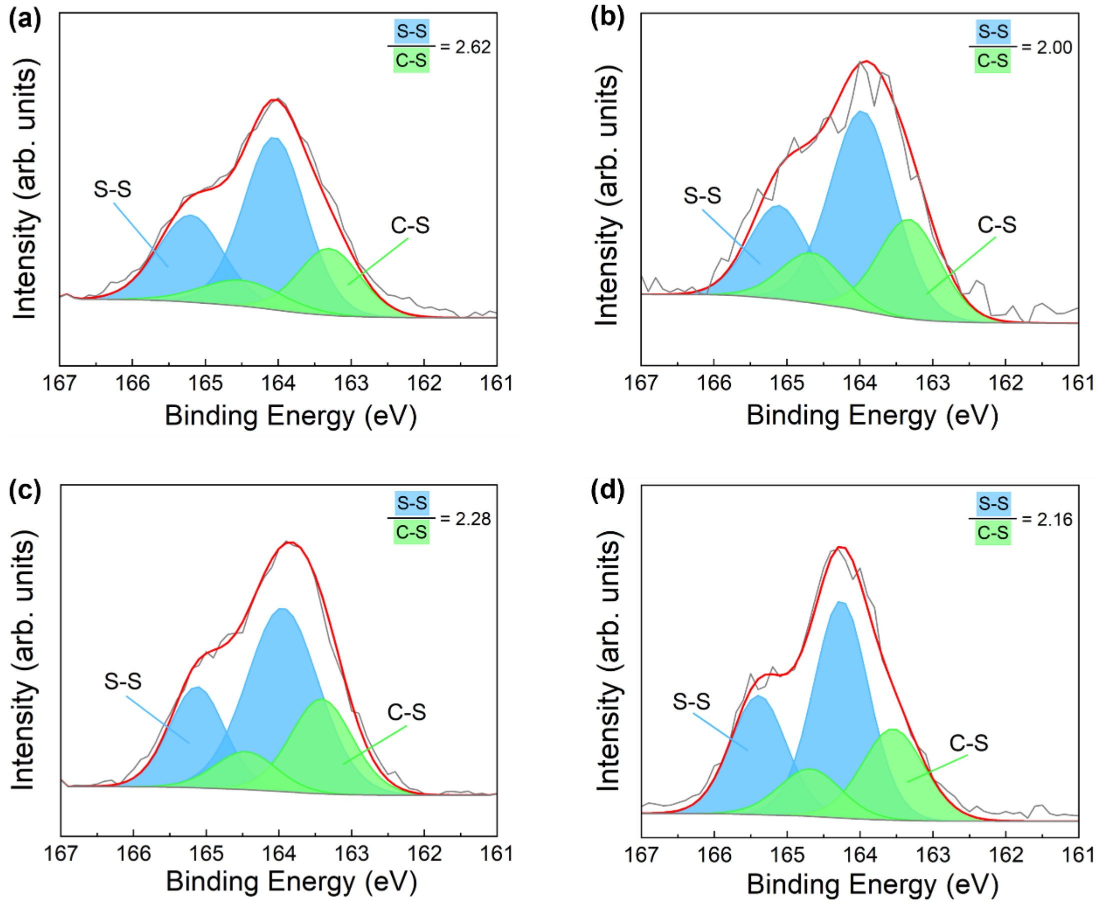

Supplementary Fig. 28 XPS spectra of S 2*p* signals of SOS4. (a) SOS4-1; (b) SOS4-2; (c) SOS4-3; (d) SOS4-4.

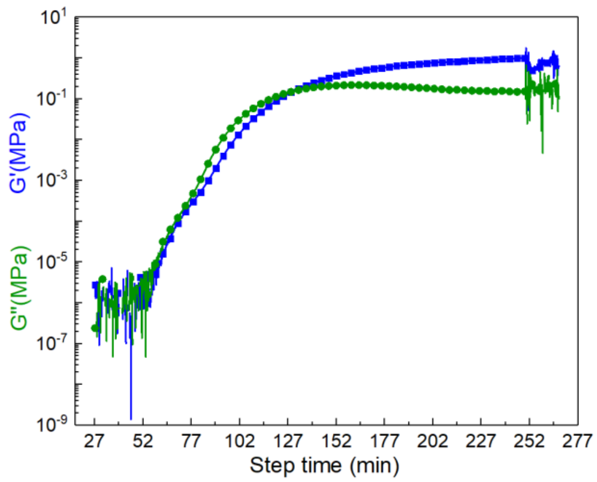

Supplementary Fig. 29 Time sweep result of DCPD1 sample with a sulfur feed ratio of 2 at 1 rad/s, 0.2% and 160 °C.

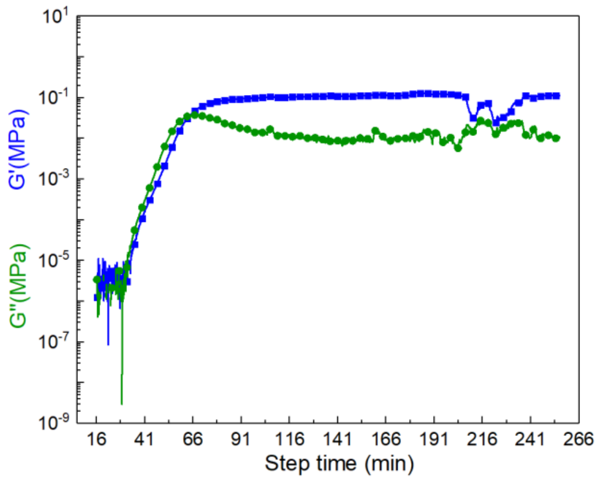

Supplementary Fig. 30 Time sweep results of DCPD2 sample with a sulfur feed ratio of 2 at 1 rad/s, 0.2% and 170 °C.

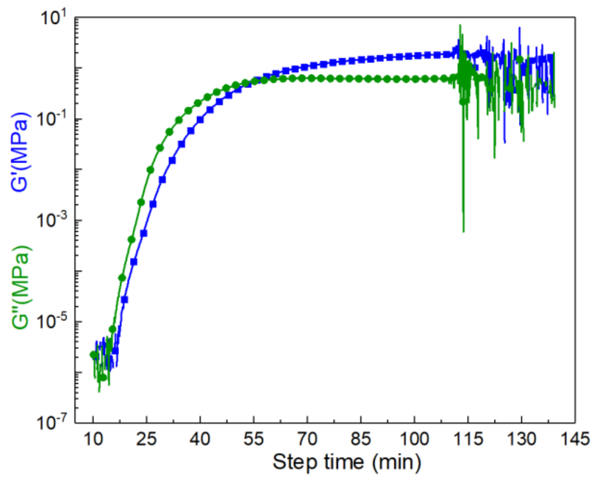

Supplementary Fig. 31 Time sweep result of DCPD3 sample with a sulfur feed ratio of 2 at 1 rad/s, 0.2% and 180 °C.

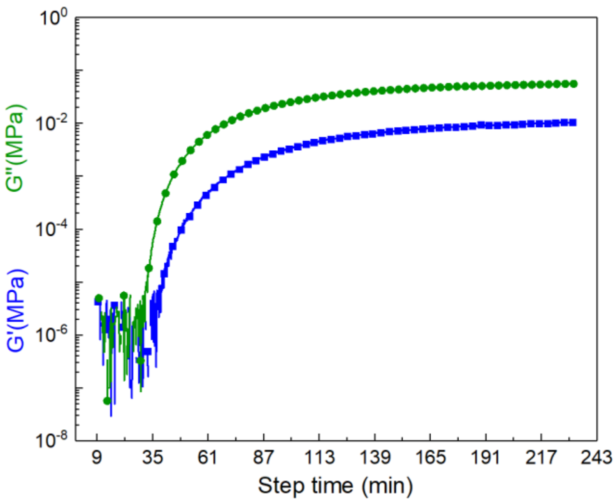

Supplementary Fig. 32 Time sweep result of DCPD4 sample with a sulfur feed ratio of 4 at 1 rad/s, 0.2% and 160 °C.

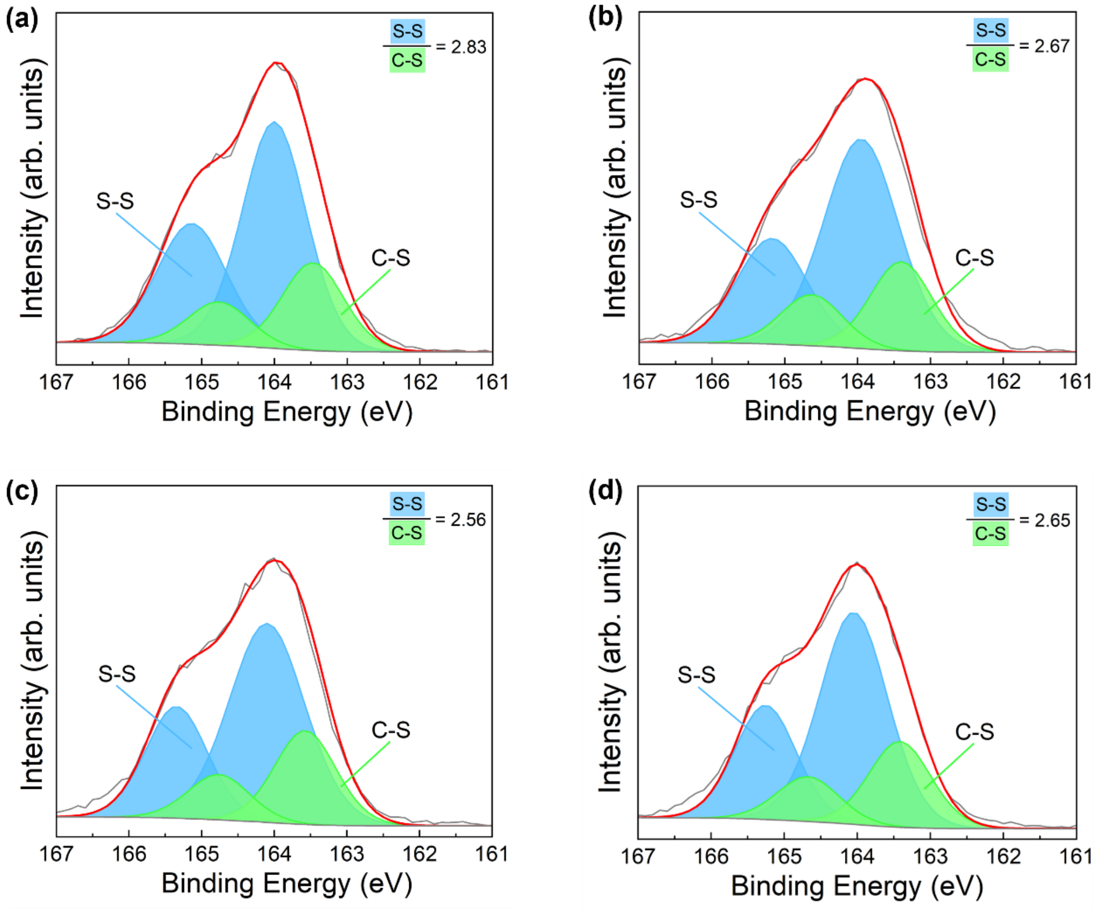

Supplementary Fig. 33 The high-resolution S 2*p* XPS spectra of poly(DCPD-*r*-S) at different inverse vulcanization time: (a) 80 min; (b) 130 min, (c) 180 min and (d) 240 min.

Supplementary Fig. 33 shows the XPS spectra of inverse vulcanization product of DCPD with different reaction time and the sulfur rank of the samples are calculated from deconvolution of S 2*p* signals. Interestingly, the poly(S-*r*-DCPD) has a sulfur rank of 7.66, 7.34, 7.12 and 7.30 at 80, 130, 180 and 240 min, respectively, which is higher than poly(S-*r*-DIB) and poly(S-*r*-SOS) (6–7). The high sulfur rank could be ascribed to incomplete conversion of alkenyl groups with steric hindrance which consume sulfur chains slowly as evidenced by as the decreased sulfur rank inverse vulcanization proceeded. Therefore, the trace loss of G' and gelation at extended curing is supposed to ascribed to the nature of stability within DCPD instead of short sulfur chains unfavorable for side reaction.

Supplementary Table 4. Inverse vulcanization of mono-alkenes and an aliphatic ester for mechanistic investigation.

Sample	Monomer Structures	S_8 (g)	Monomers (g)	sulfur feed ratio [a]	Temperature (°C)	Time (h)
αMStS1		0.71	1.31	2	160	0.5~4 (Sampled by every half hour)
αMStS2		0.90	1.11	3	160	0.5~4 (Sampled by every half hour)
αMStS3	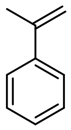	1.04	0.96	4	160	0.5~4 (Sampled by every half hour)
αMStS4	a-Menthylstyrene (aMSt)	0.70	1.30	2	140	1~7(Sampled every one hour)
αMStS5		0.73	1.30	2	150	1~7(Sampled every one hour)
αMStS6		0.71	1.30	2	160	1~7(Sampled every one hour)
4-MStS	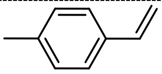 4-Methylstyrene (4-MSt)	0.70	1.30	2	160	24
APES	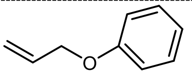 Allyl phenyl ether (APE)	0.65	1.35	2	160	24
EODS	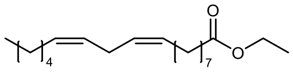 Ethyl octadeca-9,12-dienoate (EOD)	0.91	1.10	4	180	4
SA	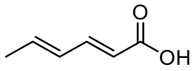 Sorbic acid (SA)	0.93	1.07	2	180	4

[a] The sulfur feed ratio is the molar ratio of sulfur over alkene groups ([S]/[C=C]) in the feedstocks.

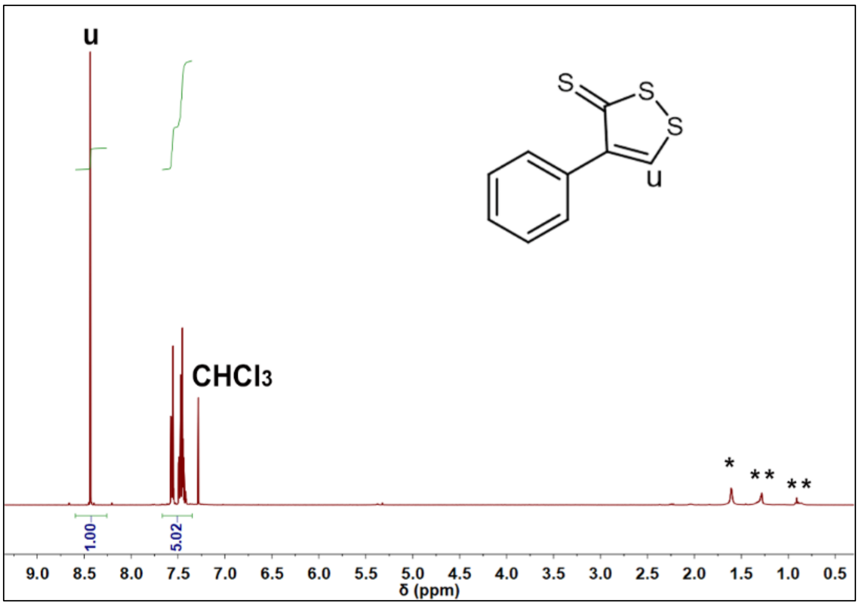

Supplementary Fig. 34 ^{1}H NMR spectrum of 1,2-dithiol-2-phenyl-3-thione separated from inverse vulcanization product of αMSt using $CDCl_3$ as the solvent.

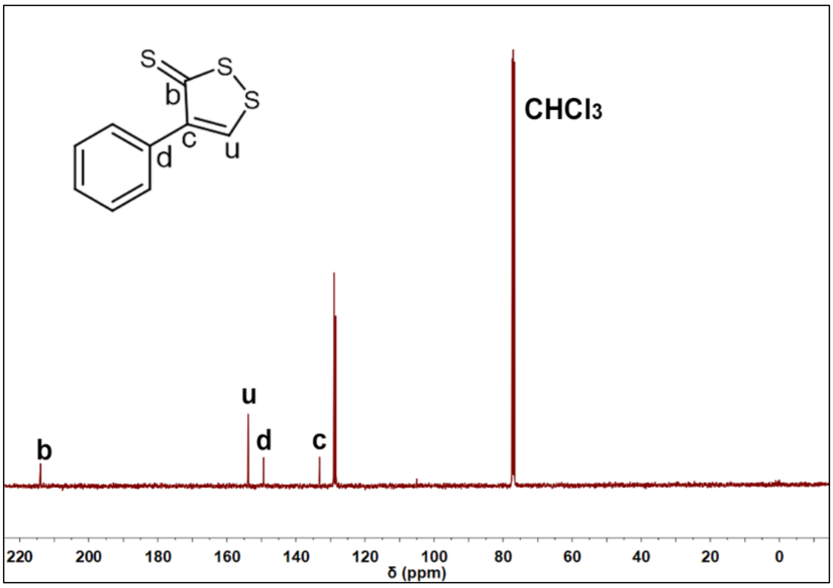

Supplementary Fig. 35 ^{13}C NMR spectrum of 4-phenyl-1,2-dithiol-3-thione separated from inverse vulcanization product of αMSt. The solvent is $CDCl_3$ using $CDCl_3$ as the solvent.

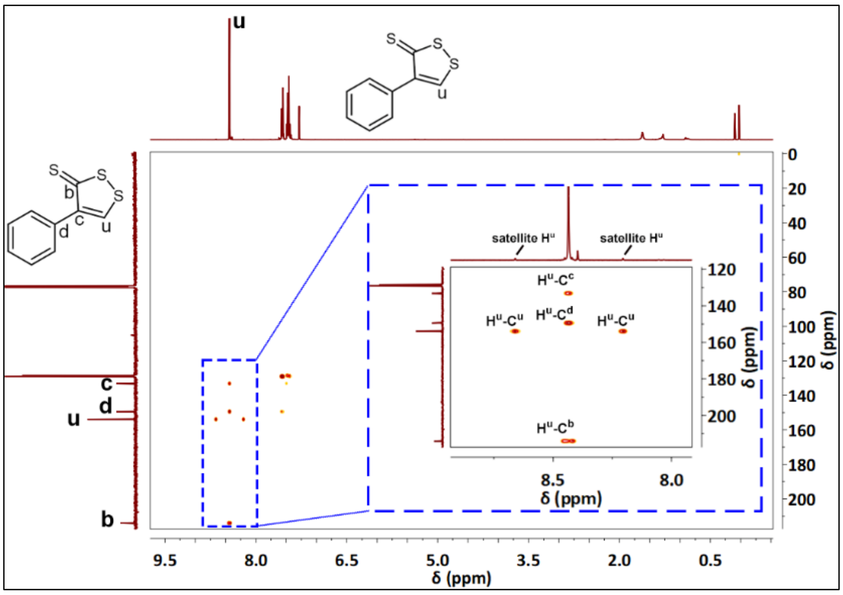

Supplementary Fig. 36 HMBC spectrum of 4-phenyl-1,2-dithiol-3-thione separated from inverse vulcanization product of αMSt with zoom-in view display the long-range correlation signals between proton and carbon signals from DTR rings (solvent: $CDCl_3$).

Supplementary Figs. 34–36 illustrate the ^1H, ^{13}C and HMBC spectra of 4-phenyl DTT. It can be seen that the strong proton signal at 8.5 ppm is correlated with not only thiocarbonyl carbon (C^b) but also carbons with chemical shifts around 130 to 150 ppm, which can be ascribed to aromatic carbon and conjugated alkenes carbons. The correlation and the significant de-shielding effect posed on both C^c and C^u along with proton (H^u) on it suggest that the atoms belong to alkenes connected with electron-withdrawn groups and aromatic groups. Therefore, the structure of DTT is validated, which is consistent with results reported in the literature.[6]

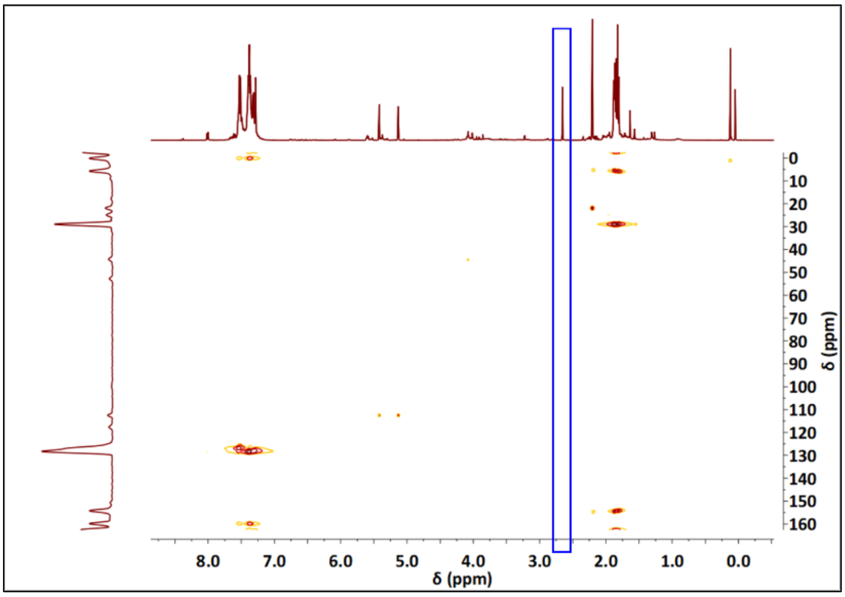

Supplementary Fig. 37 HSQC spectrum of αMSt samples reacted for 1 hour at 160 °C with a sulfur feed ratio of 2. The blue box highlights the area where signal of proton signal with a chemical shift of 2.6 ppm was expected to appear (solvent: $CDCl_3$).

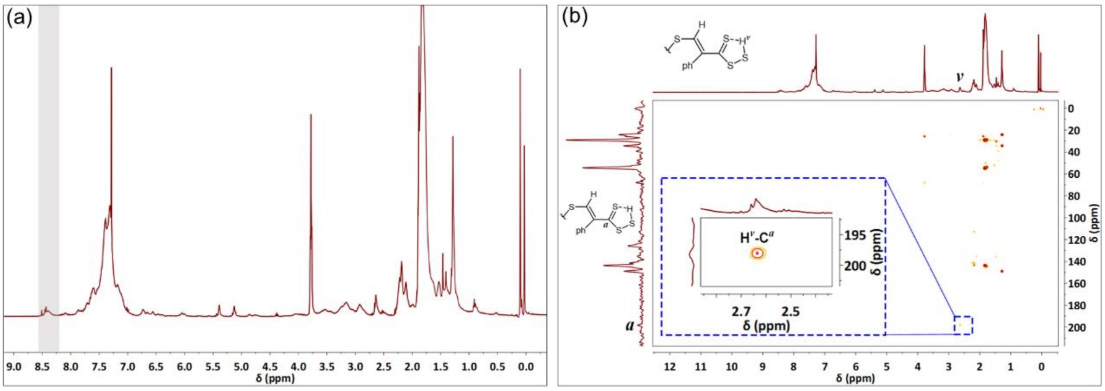

Supplementary Fig. 38 (a) ^{1}H NMR and (b) HMBC spectra of the soluble fraction of DIBS1-4 sample. The shadow highlights the DTT signals, and the zoom-in view in the HMBC spectrum displays the long-range correlation signals between proton and carbon signals belonging to CDTA moiety (solvent: $CDCl_3$).

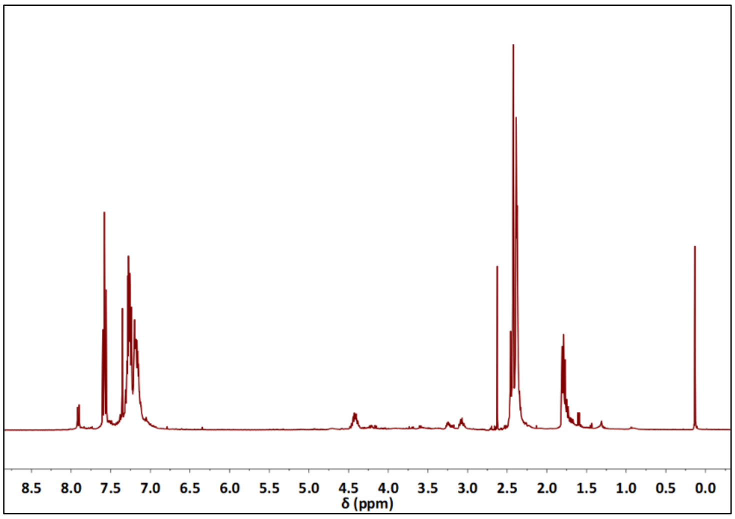

Supplementary Fig. 39 ^{1}H NMR spectra of 4-MStS sample using $CDCl_3$ as the solvent.

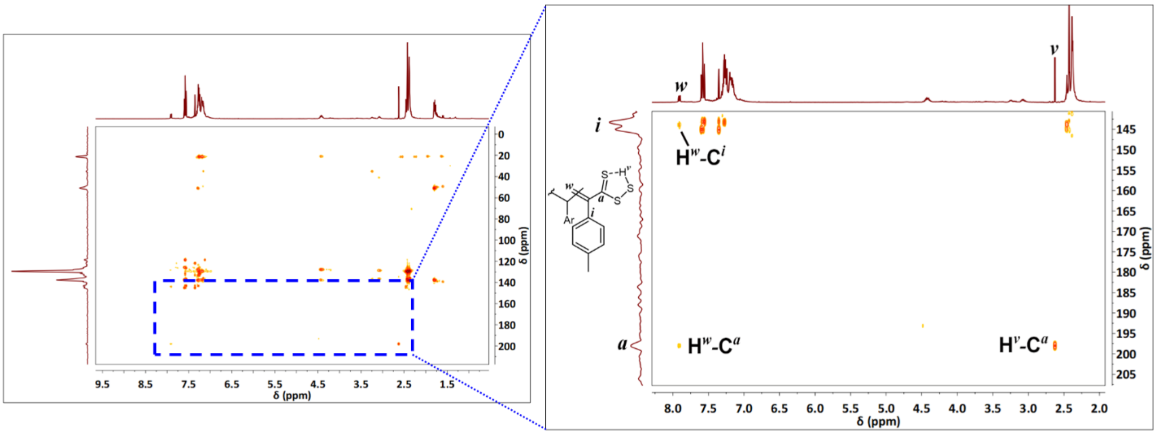

Supplementary Fig. 40 HMBC spectra of 4-MStS sample. (Ar = 4-methylphenyl) The shadow highlights proton signals correlating with C^a signal at low fields revealed by the HMBC spectrum (solvent: $CDCl_3$).

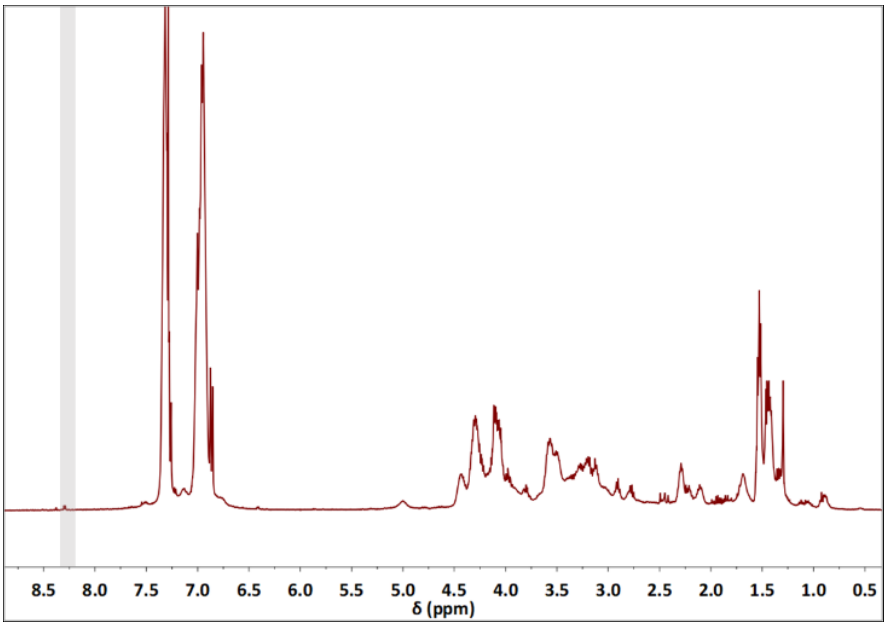

Supplementary Fig. 41 ^{1}H NMR spectrum of APES sample. The shadow highlights proton signals ascribed to DTR structure (solvent: $CDCl_3$).

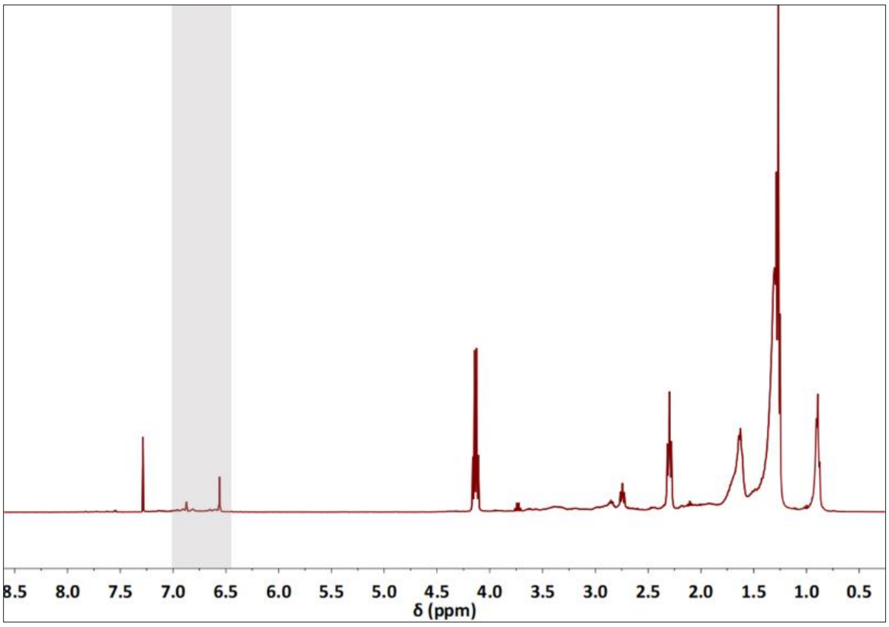

Supplementary Fig. 42 ^{1}H NMR spectrum of EODS sample. The shadow highlights proton signals ascribed to protons signals belonging to 2,5-substituted thiophene structures (solvent: $CDCl_3$).

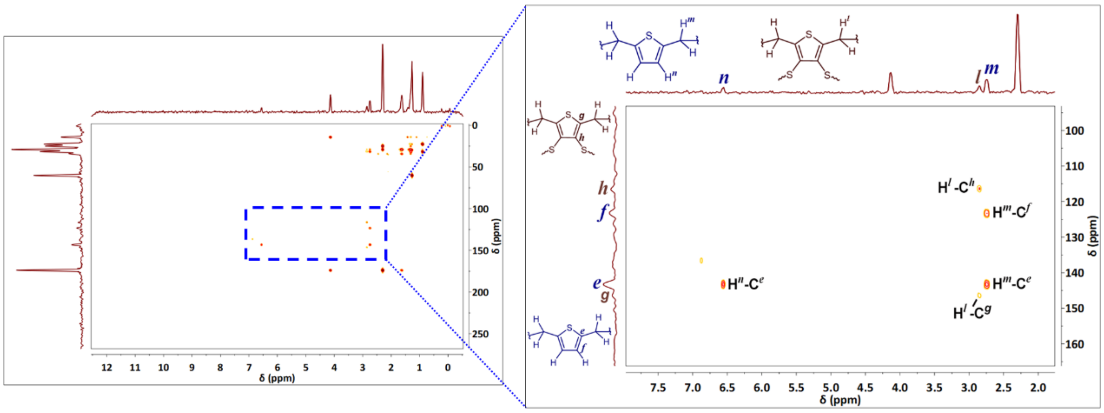

Supplementary Fig. 43 HMBC spectrum of EODS sample with its zoom-in view highlighting the long-range correlation signals between proton and carbon signals belonging to 2,5-substituted thiophene moieties (solvent: $CDCl_3$).

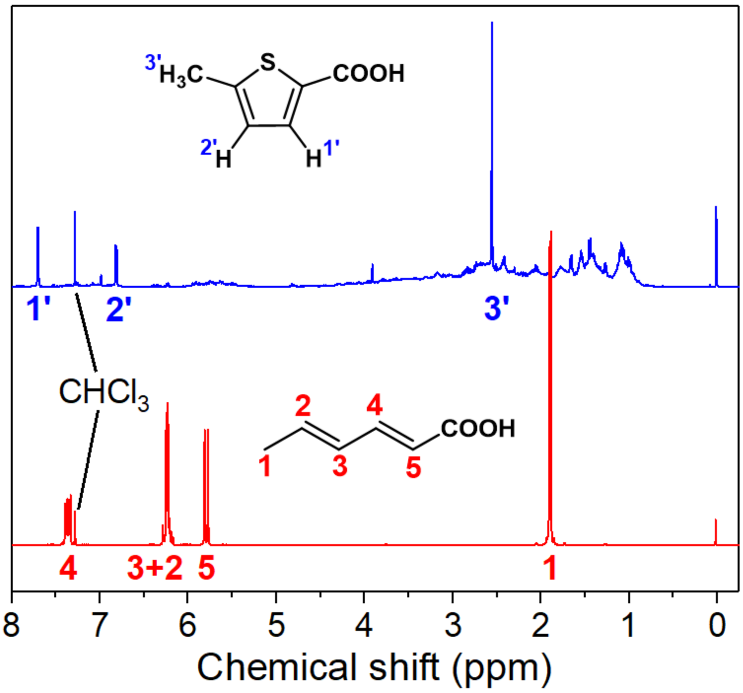

Supplementary Fig. 44 ^1H NMR spectrum of P(S-SA) using $CDCl_3$ as the solvent. The proton integral ratios $H^{1'}$:$H^{2'}$: $H^{3'}$ = 1 : 1: 3.5.

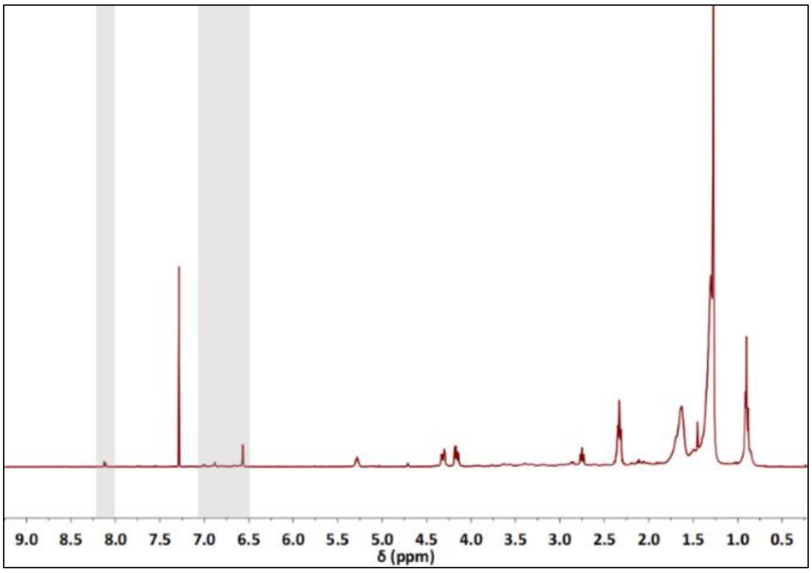

Supplementary Fig. 45 ^{1}H NMR spectrum of soluble fraction of SOS4-4 sample using $CDCl_3$ as the solvent. The shadow highlights the signals belonging to DTR-like structure and substituted thiophene moieties.

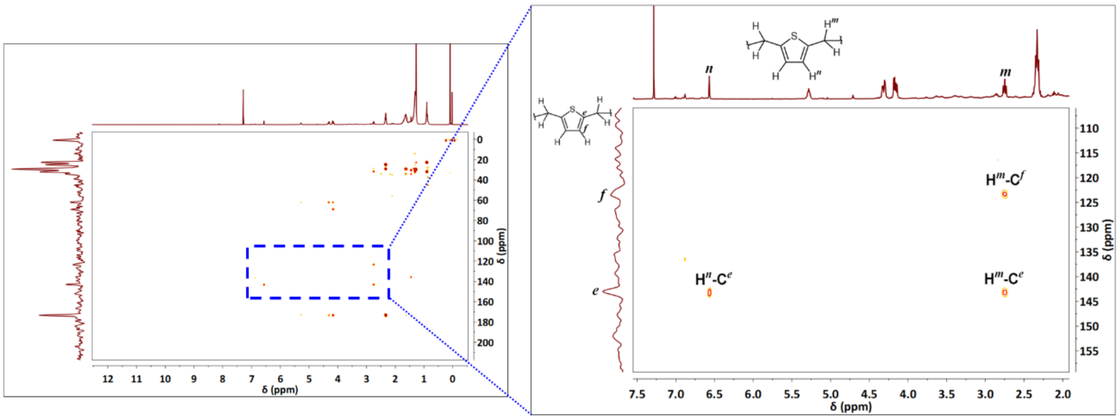

Supplementary Fig. 46 HMBC spectrum of soluble fraction of SOS4-4 sample using $CDCl_3$ as the solvent.. The zoom-in view in the HMBC spectrum displays the long-range correlation signals between proton and carbon signals belonging to 2,5-substituted thiophene moiety.

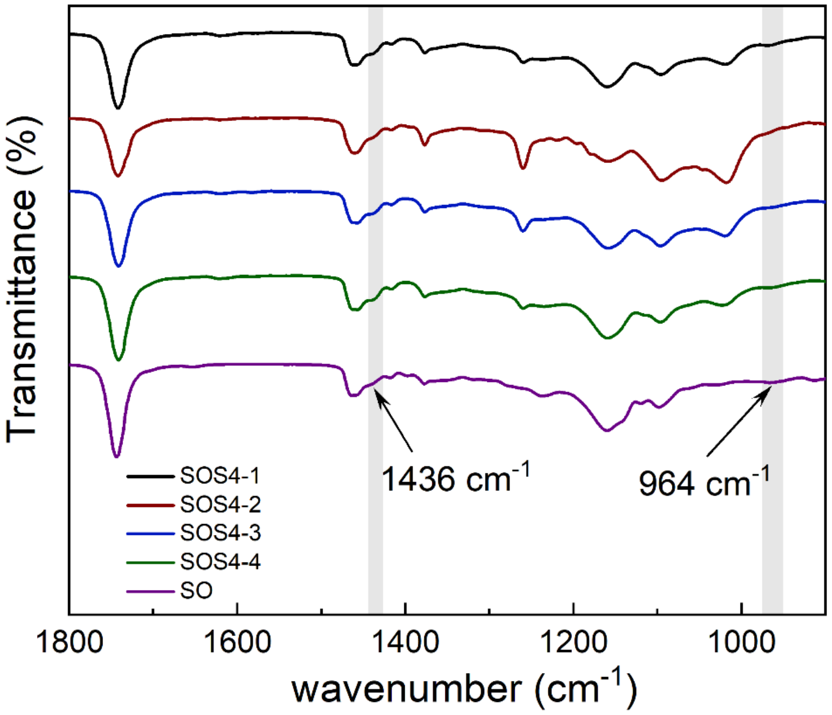

Supplementary Fig. 47 FT-IR spectrum of soluble fraction of SOS4-1, SOS4-2, SOS4-3 and SOS4-4 samples. The shadow highlights the adsorption arising from vibration of C=C bond in substituted thiophene moieties.

Supplementary Reference:

1. Coran AY. Vulcanization: Conventional and Dynamic. *Rubber Chem. Technol.* **68**, 351-375 (1995).
2. Shundo A, Aoki M, Yamamoto S, Tanaka K. Effect of Cross-Linking Density on Horizontal and Vertical Shift Factors in Linear Viscoelastic Functions of Epoxy Resins. *Macromolecules* **54**, 9618-9624 (2021).
3. Zaccone A, Terentjev EM. Disorder-assisted melting and the glass transition in amorphous solids. *Phys. Rev. Lett.* **110**, 178002 (2013).
4. Wang F, Saeki S, Yamaguchi T. Temperature and pressure dependence of thermal expansion coefficient and thermal pressure coefficient for amorphous polymers. *Polymer* **38**, 3485-3492 (1997).
5. Ding Y, Sokolov AP. Comment on the dynamic bead size and Kuhn segment length in polymers: Example of polystyrene. *J. Polym. Sci. B Polym. Phys,* **42**, 3505-3511 (2004).
6. Onose Y, Ito Y, Kuwabara J, Kanbara T. Tracking side reactions of the inverse vulcanization process and developing monomer selection guidelines. *Polym. Chem.* **13**, 5486-5493 (2022).
